# Continued Stabilization of the Nuclear Higher-Order Structure of Post-Mitotic Neurons In Vivo

**DOI:** 10.1371/journal.pone.0021360

**Published:** 2011-06-23

**Authors:** Janeth Alva-Medina, Apolinar Maya-Mendoza, Myrna A. R. Dent, Armando Aranda-Anzaldo

**Affiliations:** Laboratorio de Biología Molecular y Neurociencias, Facultad de Medicina, Universidad Autónoma del Estado de México, Toluca, Estado de México, México; UT Southwestern Medical Center, United States of America

## Abstract

**Background:**

Cellular terminal differentiation (TD) correlates with a permanent exit from the cell cycle and so TD cells become stably post-mitotic. However, TD cells express the molecular machinery necessary for cell proliferation that can be reactivated by experimental manipulation, yet it has not been reported the stable proliferation of any type of reactivated TD cells. Neurons become post-mitotic after leaving the ventricular zone. When neurons are forced to reenter the cell cycle they invariably undergo cell death. Wider evidence indicates that the post-mitotic state cannot solely depend on gene products acting in *trans*, otherwise mutations in the corresponding genes may lead to reentry and completion of the cell cycle in TD cells, but this has not been observed. In the interphase, nuclear DNA of metazoan cells is organized in supercoiled loops anchored to a nuclear nuclear matrix (NM). The DNA-NM interactions define a higher-order structure in the cell nucleus (NHOS). We have previously compared the NHOS of aged rat hepatocytes with that of early post-mitotic rat neurons and our results indicated that a very stable NHOS is a common feature of both senescent and post-mitotic cells in vivo.

**Principal Findings:**

In the present work we compared the NHOS in rat neurons from different post-natal ages. Our results show that the trend towards further stabilization of the NHOS in neurons continues throughout post-natal life. This phenomenon occurs in absence of overt changes in the post-mitotic state and transcriptional activity of neurons, suggesting that it is independent of functional constraints.

**Conclusions:**

Apparently the continued stabilization of the NHOS as a function of time is basically determined by thermodynamic and structural constraints. We discuss how the resulting highly stable NHOS of neurons may be the structural, non-genetic basis of their permanent and irreversible post-mitotic state.

## Introduction

The hallmark of cellular terminal differentiation (TD) is a permanent exit from the cell cycle and so TD cells become stably post-mitotic. Paradoxically, TD cells express the molecular machinery necessary for cell proliferation that can be reactivated by different experimental manipulations [1], [2]. So far it has not been reported the stable proliferation of any type of TD cells as full division of reactivated TD cells rarely occurs and the newly formed cells die in the short term. Indeed, TD reactivated cells more often suffer immediate or delayed cell death and in some particular cases become permanently arrested in the G2 phase of the first cell cycle [2].

Cortical neurons are a prime example of TD cells and in mammals they become post-mitotic after they leave the germinal centers located in the ventricular zone [3]. When TD neurons are forced to reenter the cell cycle they invariably undergo cell death [4]–[6]. This phenomenon is known as cell-cycle related neuronal death (CRND). Recently it has been shown that Cdk5 a nontraditional cyclin-dependent kinase very active in post-mitotic neurons is a potent cell-cycle suppressor that arrests the neuronal cell cycle by a non-catalytic mechanism [7]. Absence of Cdk5 in the neuronal nucleus leads to loss of cell cycle control and death of post-mitotic neurons, suggesting that Cdk5 has a critical neuron-protective function by suppressing the reentry into the cell cycle that leads to neuronal death [7], [8].

Since eliminating the activity of inhibitors of cyclin-dependent kinases leads to reentry into the cell cycle of TD muscle cells even in the absence of growth factors, this has led to the suggestion that the post-mitotic state is an active state mediated by specific gene products [1], [9]. However, wider evidence indicates that the stability of the post-mitotic state cannot be determined only by the continued action of soluble gene products acting in *trans*, otherwise spontaneous or provoked mutations in the corresponding genes may lead to eventual reentry and successful completion of the cell cycle by formerly TD cells, but this has not been observed. Indeed, the post-mitotic state of TD cells such as cortical neurons and cardiomyocytes is so stable that no bona fide tumors originating in such TD cells have been described. Indeed, primary brain tumors arise from the glial cells that preserve a proliferating potential or from precursor cells such as retinoblasts in children, but so far no malignancy of post-mitotic neurons has occurred spontaneously or been induced by carcinogens in the adult cortex [10]. Primary heart tumors are extremely rare and so far such tumors always derive from heart cells which are not cardiomyocytes [11]. Moreover, organisms mainly constituted by post-mitotic cells do not develop cancer as shown in adult *Drosophila melanogaster*, a post-mitotic organism save for its germ cells, in which tumors may only arise before the larval stage, when the somatic cells are not yet TD and so preserve a proliferating potential. Indeed, adult flies subject to mutagenic radiation may die but do not develop cancer [12]–[15]. These facts suggest that there is no set of somatic mutations able to cancel the post-mitotic state.

In the interphase, nuclear DNA of metazoan cells is organized in supercoiled loops anchored to a nuclear compartment known as the nuclear matrix (NM) that is a non-soluble complex of ribonucleoproteins obtained after extracting the nucleus with high salt and treatment with DNase [16], [17]. The DNA is anchored to the NM by means of non-coding sequences of variable length known as matrix attachment regions or MARs. Yet there is no consensus sequence for a priori identification of MARs [18]. The DNA-NM interactions define a higher-order structure in the cell nucleus (NHOS).

Hepatocytes preserve a remarkable proliferating potential that can be elicited in vivo by partial hepatectomy leading to liver regeneration [19]. We have shown that in the nucleus of primary rat hepatocytes the average DNA loop size becomes significantly reduced as a function of animal age implying the progressive increase in the number of DNA-NM interactions. This fact correlates on the one hand with a dramatic strengthening of the NM framework and the stabilization of such DNA-NM interactions and on the other hand, with reduction of the cell proliferating potential and progression towards terminal differentiation of the hepatocytes with age [20]. We have also compared the NHOS of aged rat hepatocytes with that of early post-mitotic rat neurons, the results indicated that a very stable NHOS is a common feature of both aged and post-mitotic cells in vivo [21]. In the present work we compared the NHOS in rat neurons from different post-natal ages and our results show that the trend towards further stabilization of the NHOS continues in neurons beyond the fourth post-natal week, when the synapses in the cerebral cortex become indistinguishable for those in the adult rat brain and so the neurons are formally regarded as terminally differentiated [22]–[24]. This phenomenon occurs in absence of overt changes in the post-mitotic state and transcriptional activity of neurons, suggesting that the continued stabilization of the NHOS as a function of time is basically determined by thermodynamic and structural constraints. We discuss how the resulting highly stable NHOS of neurons may be the non-genetic basis of their permanent and irreversible post-mitotic state.

## Results

### High stability of the NHOS of post-mitotic neurons when compared to that of glial cells.and hepatocytes

The rat cerebral cortex contains both neurons and glial cells. The classical method by Thompson [25] designed for the isolation of neuronal nuclei separates two nuclear populations: N1 mostly constituted by neuronal nuclei according to old morphological criteria and N2 relatively enriched for glial-cell nuclei on morphological grounds. NeuN/Fox-3 is a protein specific of neuronal nuclei [26], [27] and so labelling with anti-NeuN mAb is the current standard for reliable identification of neurons and neuronal nuclei. In newborn rats (P0) the glial cells constitute a small percentage of the brain cells [3], [28] hence there is no point in separating N1 and N2 populations (Table 1). However, using anti-NeuN for identifying the true neuronal nuclei, it is clear that from post-natal day 7 onwards there is a significant presence of glial cell nuclei in both N1 and N2 populations (Table 1).

**Table 1:** Percentage of neuronal and glial nuclei and nucleoids.

Post-natal age	Nuclei				Nucleoids			
	Neurons		Glia		Neurons		Glia	
	N1	N2	N1	N2	N1	N2	N1	N2
P0	83.7±4.5	---	16.3±4.5	---	90.6±1.5	---	9.4±1.5	---
P7	81.9±2.1	73.0±9.4	18.1±2.1	27.0±9.4	93.7±3.2	91.9±1.2	6.3±3.2	8.1±1.2
P80	80.0±1.5	74.3±4.0	20.0±1.5	25.1±4.0	93.7±2.4	89.2±5.2	6.3±2.4	10.8±5.1
P540	78.3±13.0	81.5±10.7	21.7±13.0	18.5±10.7	95.3±1.9	89.4±2.7	4.7±1.9	10.6±2.7

Percentage of neuronal and glial nuclei, and of neuronal and glial nucleoids in the N1 and N2 fractions, as determined by immunofluorescence with the neuron-specific anti-NeuN mAb, in samples from new-born (P0), 7-day-old (P7), adult (P80) and old (P540) rats.

The DNA loops plus the NM constitute a nucleoid, a very large nucleoprotein aggregate generated by lysis of nuclei at pH 8.0 in non-ionic detergent and the presence of high salt concentration. In nucleoids the DNA remains essentially intact but it lacks the nucleosome structure because of the dissociation of histones and other chromatin proteins; yet the naked DNA loops remain topologically constrained and supercoiled being attached to the NM [29], [30]. Nucleoids are a standard preparation for evaluating the NHOS. After treating the N1 and N2 populations for extraction of nucleoids the resulting preparations are highly enriched in NeuN-positive neuronal nucleoids while the presence of NeuN-negative nucleoids from glial cells is significantly reduced, indicating that the glial-cell nuclei are poorly resistant to the NM-extraction conditions (Table 1). This fact underlines the overall higher stability of the NHOS from neurons when compared to that of glial cells. Moreover, we have recently shown that NeuN/Fox-3 is an intrinsic component of the neuronal NM and so it is a reliable marker of the neuronal NHOS [31].

**Figure 1:**
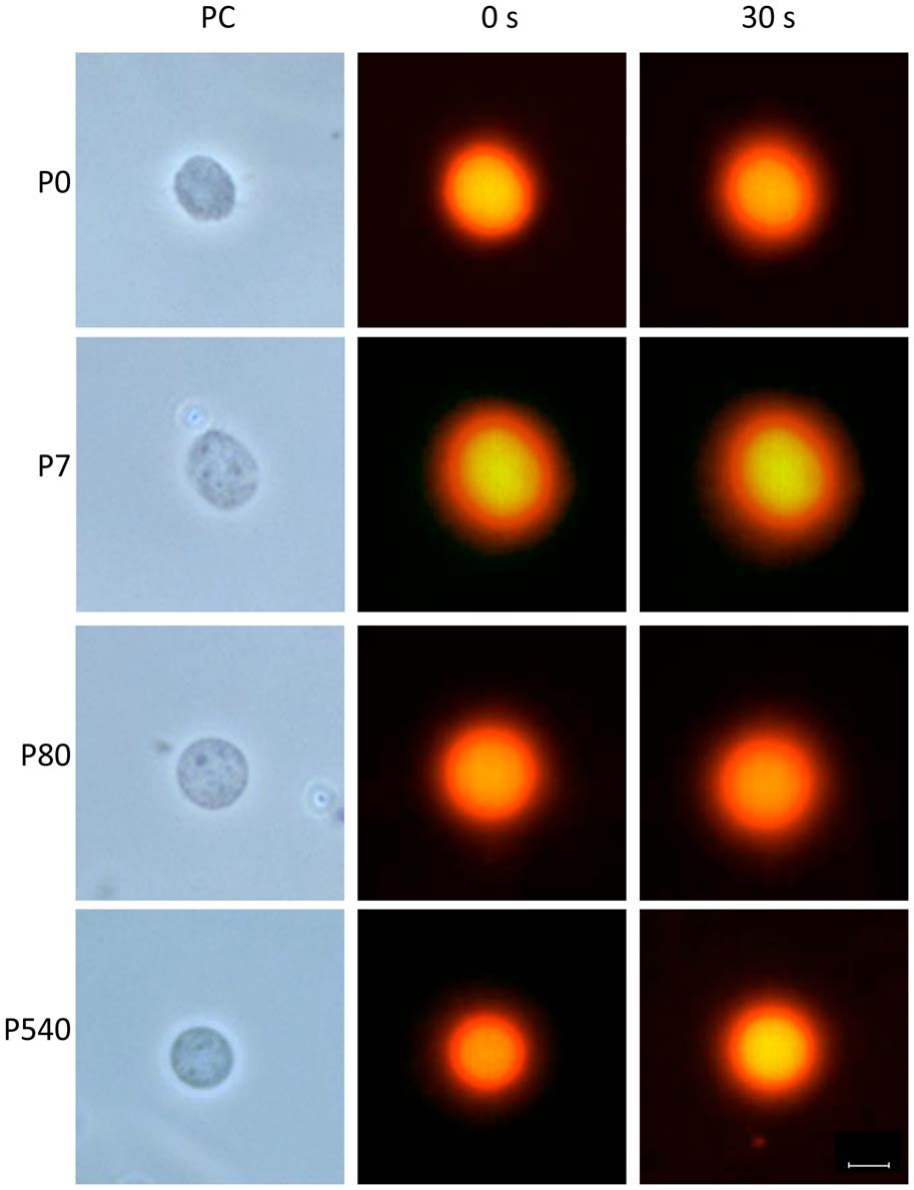
Neuronal nucleoids from the cerebral cortex of rats of different post-natal ages. Nucleoids were treated with 80 µg/ml of ethidium bromide for inducing the unwinding of the DNA loops that form a halo around the NM. 0 s and 30 s correspond to the first and last micrograph from a 30 s real-time video (see Videos S1, S2, S3, S4) that shows the stability of the neuronal NM and the DNA-NM interactions at all ages studied (P0, P7, P80 and P540 days post-partum), since in no case the NM framework was affected by the unwinding of loop DNA nor the resulting halos were distorted by such unwinding at a difference of what is observed in the case of nucleoids from hepatocytes of P0 and P7 rats (see the corresponding Videos S5, S6). PC: phase contrast. Bar = 10 µm.

Nucleoids are also known as nuclear halos since exposure of such structures to DNA-intercalating agents like ethidium bromide (EB) leads to unwinding of the DNA loops that form a fluorescent DNA halo around the NM periphery. The EB acts as a molecular lever causing the unwinding of loop DNA and this process induces strong tearing forces that impinge upon the NM as the DNA rotates and expands during unwinding. Thus the stability of the DNA-NM interactions and of the NM itself can be evaluated by titrating the response of the nucleoids to increasing concentrations of EB since weak DNA-NM interactions are eliminated at lower EB concentrations. Hence, exposing the corresponding nucleoids to a high concentration of EB (80 µg/ml) leads to maximum halo expansion and under such conditions any significant change in the distribution of the DNA-NM interactions or any significant weakness in the strength of such interactions is revealed as distortions and heterogeneities in the resulting halo surrounding the NM. Moreover, a weak NM can be fractured by the tearing forces resulting from DNA loop unwinding an expansion [20]. After exposure to high [EB] the nucleoids from neurons of the four post-natal ages studied (P0, P7, P80, P540 days) display well defined DNA halos that surround the undisturbed NM framework (Fig. 1 and Videos S1, S2, S3, S4). This is in contrast with nucleoids from P0 and P7 hepatocytes that are destroyed by the resulting EB-induced DNA unwinding (Videos S5, S6) while only the nucleoids from P80 and P540 hepatocytes are able to withstand the effects of EB-induced DNA unwinding, in a similar fashion as the neuronal nucleoids (Videos S7, S8).

**Figure 2:**
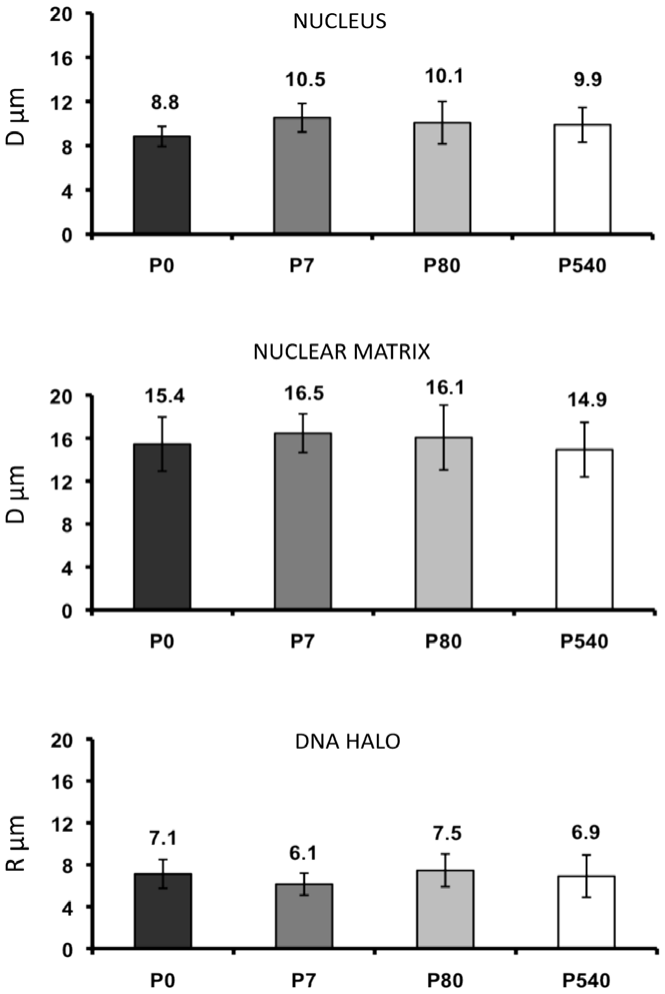
Average diameter (D) of the nucleus and the NM, and average radius (R) of the corresponding nucleoid-DNA halo from P0, P7, P80 and P540 rat neurons. Bars represent the corresponding standard deviations of measurements carried out under phase contrast (nuclei and NM) and fluorescent (DNA halo) microscopy at 40×. See Table 2 for statistical analysis of data, n = 50 for each set.. The data sets for P7 and P540 were taken from our previous work [21]. The corresponding average DNA loop size can be deduced from the measurement of the DNA halos as it has been previously described [20]: P0 = 42 kbp±4.02; P7 = 36 kbp±3.05; P80 = 44 kbp±4.6; P540 = 40.6 kbp±5.9.

We determined the average diameter of both nucleus and NM in neurons from all post-natal ages studied (Fig. 2 and Table 2). The results indicate that the NM diameter is greater than the corresponding nuclear diameter at all ages studied, suggesting that the neuronal NM is resilient as it has been observed in lower eukaryotes [32]. The average DNA halo size has been correlated with the average DNA-loop size, therefore by measuring the average DNA-halo radius after maximum halo expansion (Fig. 1 and Fig. 2) it is possible to estimate the average DNA-loop size [20], [33], [34]. The results indicate that there is no real significant change in the average DNA loop size in neurons from maturity to old age (Fig. 2 and Table 2). This is in contrast to what we have observed in hepatocytes in which the average DNA loop size is reduced by 36% from P80 (98 kbp) to P540 (60 kbp) [20]. Nevertheless, the average DNA loop size in neurons from all ages studied is significantly shorter (≤44 kbp) than that in aged P540 hepatocytes.

**Table 2:** Analysis of significance for data in Fig. 2.

T VALUE FROM STUDENT T TEST			
SIZE	NP0 & NP7	NP7 & NP80	NP80 & NP540
NUCLEUS	0.0001Significance	0.1732No significance	0.5877No significance
NUCLEAR MATRIX	0.0243No significance	0.4438No significance	0.0448No significance
DNA HALO	0.0001Significance	0.0001Significance	0.1283No significance

Student's t-test values for establishing significant differences in either the size of the nucleus, nuclear matrix or DNA halo, between neurons from different ages: new born (P0), seven days (P7), adult (P80) and old (P540) rats. The results were established using a significance level of 99% (P<0.01).

### Kinetics of nucleoid DNA digestion in samples from post-mitotic neurons of different ages

**Figure 3:**
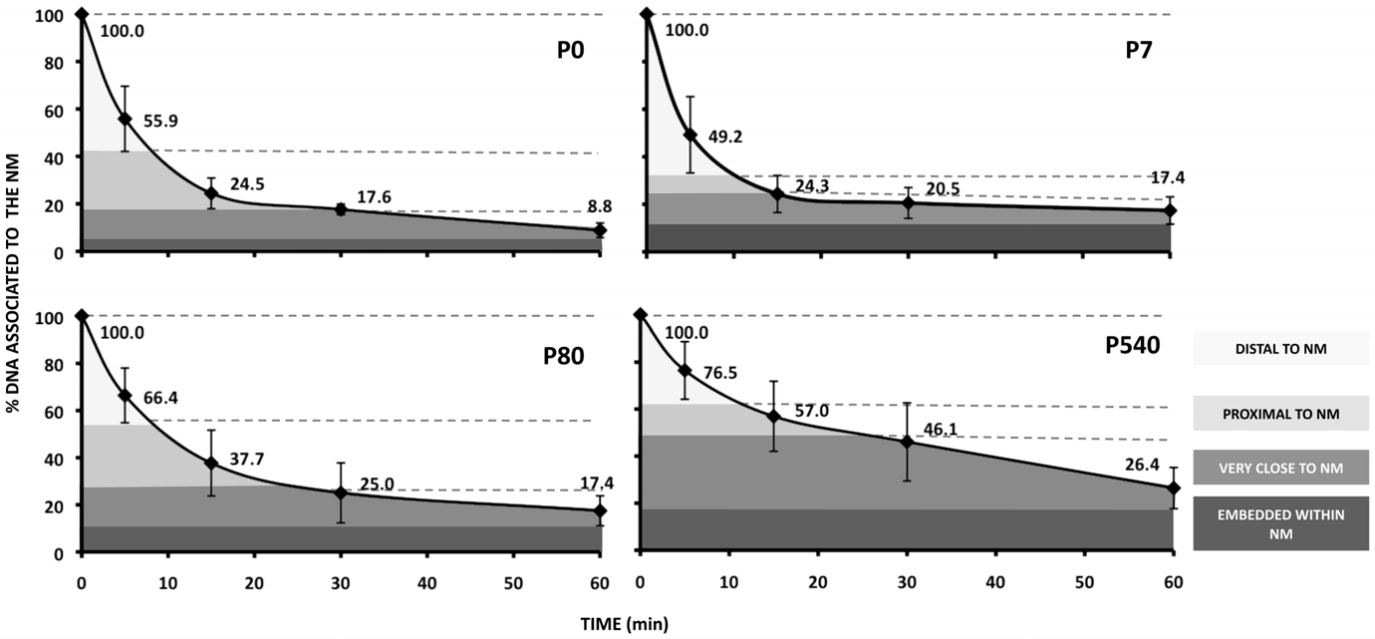
Kinetics of loop DNA digestion with DNase I in nucleoids from P0, P7, P80 and P540 rat neurons. Nucleoids were treated with DNase I at 0.92 U/ml. Each time-point value corresponds to the average of independent experiments using separate animals as the source of nucleoids (P0 n = 4; P7 n = 5; P80 n = 5; P540 n = 4). The data sets for P7 and P540 were taken from our previous work [21]. The topological zones relative to the NM for the corresponding kinetics were established considering the local slopes between pairs of time points (Table 3) and the corresponding S.D.

Treatment of neuronal nucleoids with a limited concentration of DNase I (0.92 U/ml) produces a highly reproducible kinetics of digestion of loop DNA as a function of post-natal age (Fig. 3). The naked DNA loops anchored to the NM are topologically equivalent to closed DNA circles. Under such condition the DNA molecule undergoes significant structural stress that is spontaneously dissipated by coiling the molecule upon its own axis thus achieving negative supercoiling in a similar fashion as a pulled house-telephone cord [35]. Thus the naked DNA loops display a gradient of supercoiling that goes from lower to higher from tip to base of the loop save for the fact that the structural properties of MARs are such that they also function as buffers or sinks of negative supercoiling thus avoiding maximal supercoiling at the base of the loops [36]. In nucleoid preparations the relative resistance of a given loop-DNA sequence to a limited concentration of DNase I is directly proportional to its proximity to the NM anchoring point [37], [38], two main factors determine such property: (1) steric hindrance resulting from the proteinaceous NM that acts as a physical barrier that relatively protects the naked loop DNA that is closer to the NM from endonuclease action. (2) The local degree of loop DNA supercoiling that is lower in the distal portions of the loop and higher in the regions proximal to the NM. Indeed, it is known that the DNA deeply embedded within the NM is very resistant to DNase I action and there is a fraction corresponding to some 2% of the total DNA that is basically non-digestible even when exposed to high concentrations of the enzyme [39]. Thus in a large sample of nucleoids exposed to a limited concentration of DNase I there is a consistent trend in which distal regions of the loop are digested first while the regions closer to the NM are digested later.

A typical DNA loop can be divided in four topological zones according to their relative proximity to the NM [38] defined by the local slopes between the pairs of time-points of the corresponding kinetics of digestion with DNase I (Table 3). In all cases the slopes become close to zero after 60 min digestion (Fig. 3). The results indicate that the overall kinetics of digestion with DNase I become slower as a function of neuronal age. In contrast with the coarse-grained biophysical technique for measuring the average DNA loop size (Fig. 2), this biochemical assay provides evidence that in P540 neurons a significantly larger fraction of total DNA is embedded in the NM when compared with the corresponding fraction in neurons from earlier ages, resulting in loops with a higher degree of supercoiling that slows down the overall kinetics of DNA digestion (Fig. 3).

**Table 3:** Local slopes from the age-specific kinetics of neuronal loop-DNA digestion.

Nucleoids								
	P0		P7		P80		P540	
TIME (min)	% of DNA attached to NM	S	% of DNA attached to NM	S	% of DNA attached to NM	S	% of DNA attached to NM	S
05	100.055.9	−8.8	100.049.2	−10.2	100.066.4	−6.7	100.076.5	−4.7
515	55.924.5	−3.1	49.224.3	−2.5	66.437.7	−2.9	76.557.0	−2.0
1530	24.517.6	−2.2	24.320.5	−0.3	37.725.0	−0.8	57.046.1	−0.7
3060	17.68.8	−3.4	20.517.4	−0.1	25.017.4	−0.3	46.126.4	−0.7

Local slopes (S) between time points from the corresponding kinetics of loop-DNA digestion of neuronal nucleoids from different post-natal ages. Topological zones relative to the NM (Fig. 3) were established according to such local slopes. New-born (P0), 7-day-old (P7), adult (P80), old (P540) rats.

### Positional mapping of target sequences relative to the NM in neuronal nucleoids of different ages

**Figure 4:**
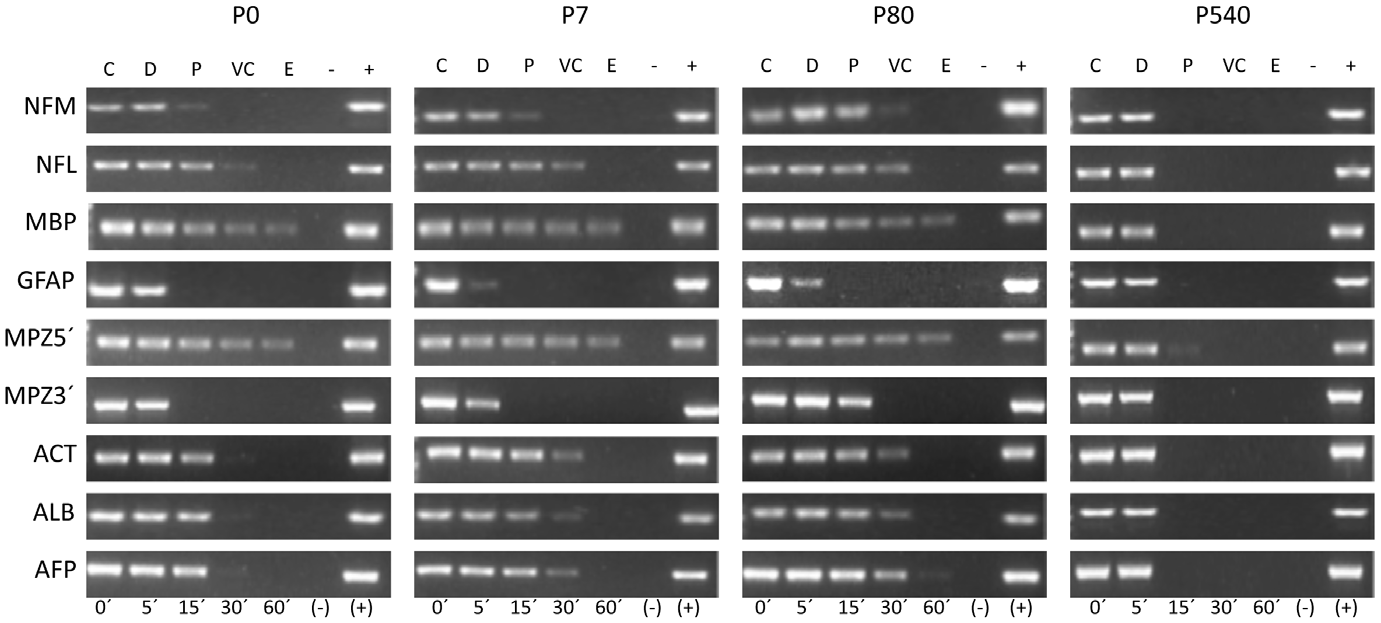
Representative amplification pattern of the chosen nine target sequences corresponding to the eight genes mapped to topological zones relative to the NM. Middle neurofilament (*NFM*), light neurofilament (*NFL*), myelin basic protein (*MBP*), glial fibrillary acidic protein (*GFAP*), myelin protein zero 5′ region (*MPZ5′*), myelin protein zero 3′ region (*MPZ3′*), beta-actin (*Act*), albumin (*Alb*), alpha-fetoprotein (*Afp*). The amplicons were scored as positive or negative by an image analysis system (Kodak 1D Image Analysis Software 3.5 system) using its default settings and thus positioned within the specific topological zones defined according to the corresponding kinetics of nucleoid-DNA digestion (Fig. 4). C = control (0′ digestion time), D = distal to the NM, P = proximal to the NM, VC = very close to the NM, E = embedded within the NM. (−) Negative control without DNA template. (+) Positive control with genomic DNA. P0, P7, P80 and P540 indicate the age of the neuronal nucleoid donors (n≥4).

It is possible to map the position of any DNA sequence relative to the NM and so to determine its location within a given DNA-loop topological zone, by coupling the kinetics of DNase I digestion (Fig. 3) with data from direct PCR amplification of the target sequences in NM-bound templates from partially digested nucleoid samples (Fig. 4). The detailed methodology for such a positional mapping has been published [37], [38], [40]. Thus, in order to establish whether the higher-order DNA structure in neurons, corresponding to the pattern of DNA loops resulting from the actual MARs attached to the NM, is the same or not throughout the post-natal period studied, we carried out a comparative positional mapping relative to the NM of nine target sequences (Table 4). Such sequences belong to eight different genes: middle neurofilament (*NFM*), light neurofilament (*NFL*), myelin basic protein (*MBP*), glial fibrillary acidic protein (*GFAP*), myelin protein zero 5′ region (*MPZ5′*), myelin protein zero 3′ region (*MPZ3′*), beta-actin (*Act*), albumin (*Alb*), alpha-fetoprotein (*Afp*) that are located in six separate rat chromosomes (Table 4) and so represent a coarse-grained sample of different chromosome territories within the cell nucleus [41]. This sample of genes differentially located within the nucleus allowed us to asses whether the positional changes relative to the NM that may occur reflect global or only local adjustments of the NHOS in time.

**Table 4:** Sets of primers for PCR and RT-PCR of target sequences.

Amplicon	Forward Primer	Reverse Primer	AmpliconLength	Access Number
	**PCR**			
NFM	5′- TCG-GCG-ATC-TCT-TCT-TTA-GCG- 3′	5′- CGG-AGC-AA-TCA-CGA-AGA-GGA- 3′	254 bp	NM_017029
NFL	5′- TCC-CCC-TTG-AGT-CCT-CTT-GA- 3′	5′- ACT-TGT-CCC-TTC-ACG-GGA-GA- 3′	249 bp	X53981
MBP	5′- CTT-CCG-AAG-GCC-TGA-TGT-GAT-3′	5′- TAA-AGA-AGC-GCC-CGA-TGG-A-3′	158 bp	K00512
GFAP	5′- TCC-AGC-CCG-TCC-CTC-AAT-AA-3′	5′- TCC-CGA-AGT-TCT-GCC-TGG-TAA-3′	418 bp	Z48978
MPZ5′	5′- CTT-GCC-CCT-ACC-CCA-GCT-AT- 3′	5′- TCT-CCT-TGG-CTG-GCT-CTC-AAT- 3′	184 bp	NM_017027
MPZ3′	5′-CCC-TGG-CCA-TTG-TGG-TTT-ACA-3′	5′-TGG-ATG-CGC-TCC-TTG-AAG-GT-3′	291 bp	NM_017027
ACT	5′- AAC-ACC-CCA-GCC-ATG-TAC-G- 3′	5′- ATG-TCA-CGC-ACG-ATT-TCC-C- 3′	254 bp	M10277
ALB	5′-GGG-ATT-TAG-TTA-AAC-AAC-TT.-3′	5′- AAA-GGT-TAC-CCA-CTT-CAT-TG- 3′	206 bp	M16825
AFP	5′-ACC-CAT-GCA-TCT-GTG-ACA-TA- 3′	5′-AGT-AAA-ATG-CAT-GTT-GCC-TG- 3′	252 bp	J02816
	**RT-PCR**			
NFM	5′- TCG-GCG-ATC-TCT-TCT-TTA-GCG- 3′	5′- CGG-AGC-AA-TCA-CGA-AGA-GGA- 3′	254 bp	NM_017029
NFL	5′- GAA-GAG-TGG-TTC-AAG-AGC-CGC-3′	5 - TCG-TGC-TTC-GCA-GCT-CAT-TC-3′	256 bp	NM_031783
ACT	5′- AAC-ACC-CCA-GCC-ATG-TAC-G - 3′	5′- ATG-TCA-CGC-ACG-ATT-TCC-C - 3′	254 bp	M10277
AFP	5′-CAG-TGA-GGA-GAA-ACG-GTC-CG- 3′	5′-ATG-GTC-TGT-AGG-GCT-CGG-CC- 3′	252 bp	NM_012493

Genomic DNA amplicons used for the positional mapping of target-gene sequences relative to the NM and amplicons for reverse transcriptase-PCR analysis of mRNA (RT-PCR) used for studying the expression of the corresponding genes. *NFM*: Neurofilament medium, chromosome location 15p12; *NFL*: Neurofilament light, chromosome 14; *MBP*: myelin basic protein, chromosome 18q11–q13; *GFAP*: Glial fibrillary acidic protein, chromosome 10q32.1; *MPZ5′*: Myelin protein zero region 5′, chromosome 13q24–q25; *MPZ3′*: Myelin protein zero region 3′, chromosome 13q24–q25; *ACT*: β-actin, chromosome 12q11; *ALB*: albumin, chromosome 14p22; *AFP*: α-fetoprotein, chromosome 14p21.

The results show that in P0 neurons most target sequences lie embedded or very close to the NM (Fig. 4 and Table 5), mimicking the trend previously reported in newborn hepatocytes for having the gene sequences in privileged locations very close to the NM [20]. However, in the P7 and P80 neurons fewer target sequences remain embedded within the NM although most gene sequences studied remain very close to the NM. Yet in the aged P540 neurons most of the target sequences have moved to locations further removed from the NM (Fig. 4 and Table 5) following the trend already described in aged P540 hepatocytes in which the gene sequences become distal from the loop anchoring points to the NM [20]. These results imply a continued adjustment of the DNA-NM interactions in time and yet the intrinsic topology of the particular DNA sequence is respected as shown by the example of the *MPZ* gene whose 5′ end is always closer to the NM than the corresponding 3′ end at all post-natal ages (Fig. 4 and Table 5).

**Table 5:** Location of target sequences within specific topological zones relative to the NM.

Topological zones relative to NM																
Amplicon	D P VC E	DD P VC E	DD P VC E	DD P VC E
	P0				P7				P80				P540			
NFM	+	+	+	−	+	+	±	−	+	+	±	−	+	±	−	−
NFL	+	+	+	±	+	+	±	−	+	+	+	±	+	±	−	−
MBP	+	+	+	+	+	+	+	+	+	+	+	±	+	+	±	−
GFAP	+	±	−	−	+	−	−	−	+	+	±	−	+	−	−	−
MPZ5′	+	+	+	+	+	+	+	+	+	+	+	+	+	±	−	−
MPZ3′	+	±	−	−	+	−	−	−	+	+	±	−	+	−	−	−
ACT	+	+	+	±	+	+	+	−	+	+	+	−	+	±	−	−
ALB	+	+	+	±	+	+	+	−	+	+	+	−	+	±	−	−
AFP	+	+	+	±	+	+	+	−	+	+	+	−	+	±	−	−

Location of the specific gene-target sequences in the topological zones relative to the NM. (D) distal to NM, (P) proximal to NM, (VC) very close to NM and (E) embedded within NM. Note that in P0 neurons most of the gene sequences were located in the embedded zone, while in the old neurons (P540) most of the gene sequences were located either in the proximal or distal zone relative to the NM. New-born (P0), 7-day-old (P7), adult (P80), old (P540) rats. (NFM) neurofilament medium; (NFL) neurofilament light; (MBP) myelin basic protein; (GFAP) glial fibrillary acidic protein; (MPZ5′) myelin protein zero region 5′; (MPZ3′) Myelin protein zero region 3′; (ACT) β-actin; (ALB) albumin; (AFP) α-fetoprotein. ± indicates that in one experiment the amplification of the target sequence was negative in the sample corresponding to the topological zone indicated (n≥4).

### Changes in the neuronal NHOS with age have no overt impact on the expression of neuron-specific genes

**Figure 5:**
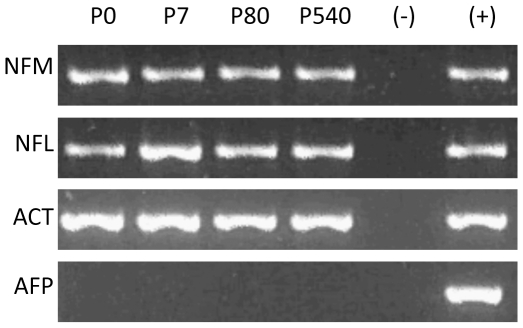
Reverse transcriptase-PCR analysis of mRNA (RT-PCR). The results show the expression of two neuron-specific genes (*NFM*, *NFL*) whose position relative to the NM in post-natal neurons was previously mapped (Table 5). *ACT* is included as control for gene expression in the cerebral cortex. *AFP* is not expressed in the cerebral cortex and it remains so. The results suggest there is no overt change in neuronal transcriptional activity with age. (−) Negative control. (+) Positive control.

Earlier reports suggested a positive correlation between proximity of a gene to the NM and active transcription [42], [43]. Yet currently there is consistent evidence that in the structural DNA loops the positioning of genes relative to the NM is independent of transcription [20], [40], [44]–[46]. Nevertheless, we performed reverse transcriptase-PCR analysis of mRNA (RT-PCR) in order to assess if the continued rearrangements of DNA loops as a function of neuronal age have an impact on genes whose normal expression is either neuron-specific or absent in the cerebral cortex and that changed their position relative to the NM in time (Table 4). The results indicate that there is no overt change in the expression of the neuron-specific genes *NFM* and *NFL* throughout the post-natal period studied, while the *AFP* gene remains consistently non-expressed in the cortex (Fig. 5), suggesting that in neurons transcriptional activity is independent of the changes in DNA-NM interactions observed along the post-natal period.

### Evidence for quantitative changes in the composition of the neuronal NM as a function of age

**Figure 6:**
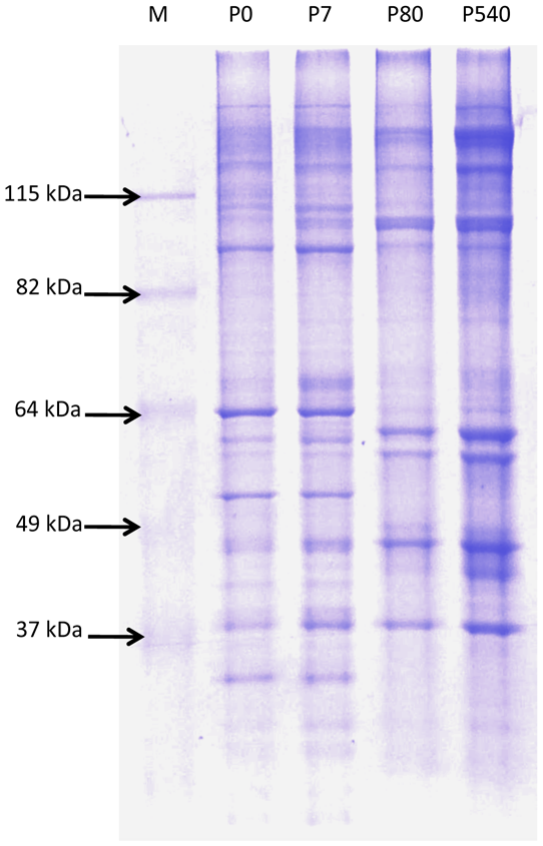
SDS-PAGE protein profiles of neuronal NM from different post-natal ages. Experimental lanes were loaded with 15 µg of the corresponding NM-protein extract. Notice the quantitative changes in the protein profiles between the early (P0, P7) and late (P80, P540) sampled ages. M, molecular weight ladder.

DNA-NM interactions occur on a grand scale and yet there is no consensus sequence for MARs [18] and a very limited number of specific proteins have been identified that participate in binding of DNA to the NM in a sequence-specific fashion [17]. These facts imply that such interactions are the result of indirect readout effects between DNA and NM proteins which are different from the direct readout interactions between transcription factors and specific DNA-functional groups [47]. It is known that quantitative and qualitative changes in the composition of the NM may occur throughout embryonic and post-natal development [16], [20], [48]. Indeed, the exact composition of the NM is a matter of debate as some four hundred proteins have been associated with this structure [49]. We carried out standard SDS-PAGE analysis of NM proteins from P0, P7, P80 and P540 neurons. The protein profiles indicate that there are quantitative changes in the composition of the NM since some proteins highly abundant in the NM from P0 and P7 neurons become significantly reduced in the NM from P80 and P540 neurons, while other proteins present at reduced levels in P0 and P7 become significantly augmented in the NM from P80 and P540 neurons (Fig. 6). Hence the quantitative modifications in the composition of the neuronal NM correlate with the increase in the DNA-NM interactions with neuronal age.

## Discussion

The fact that post-mitotic TD cells may be induced to re-enter the cell cycle but despite possessing the cell-proliferation machinery cannot successfully complete further cell divisions and very often die (as it is always the case in the case of neurons), calls for an explanation. In post-mitotic neurons certain genetic functions are actively involved in avoiding the eventual re-entry of such cells into the cell cycle as this is deleterious for the cells [7]. Yet, the notion that the post-mitotic state is achieved and sustained by continued activity of specific gene products [1], [9] is not consistent with the actual non-reversible nature of the post-mitotic state, since any process that depends on specific gene functions may be eventually reverted or avoided by spontaneous or induced mutations in the genes involved, but so far this has not been observed in the case of post-mitotic cells. This situation points towards a non-genetic mechanism as the key determinant of the post-mitotic state. Recently it was reported that DNA replication is intrinsically hindered in post-mitotic TD myotubes, independently of the method used for inducing such cells to re-enter the cell cycle and despite the lack of any evidence for the presence of a defective replication molecular-machinery in such cells [2]. Indeed, an important conclusion of that study was that the in post-mitotic cells the intrinsic obstacle to DNA replication might be structural rather than functional.

In metazoans nuclear DNA is organized in supercoiled loops anchored to a NM. The DNA-NM interactions define a NHOS that it is not static but apparently evolves towards maximum stability as a function of time [20], [50]. The post-mitotic state and the replicative senescence (RS) of aged cells are similar, potentially long-term, cellular states. However, the term RS actually implies three separate phenomena that are not causally related: there is a RS that correlates with the progressive shortening of chromosome telomeres as a function of number of cell divisions, but this phenomenon has only be observed in cells from primates [51]. A second kind of RS known as STASIS may occur prematurely as a response to diverse cellular stressors, this STASIS depends on specific genetic functions and so it can be reverted by mutation or inactivation of such genetic functions [52], [53]. There is a third kind of RS that occurs stochastically and so it increases its probability as a function of time, this seems to occur in all cells both in vitro and in vivo and it is not reversible [50], [54], [55]. A self-stabilizing model for DNA-NM interactions as a function of age, in which a stochastic but time-dependent (age-dependent) process leads to a significant increase of DNA-NM interactions, resulting in an integral and highly-stable structural system, has been proposed for explaining a common physical basis for both stochastic RS senescence and the post-mitotic state [50]. We have recently shown that aged hepatocytes and early post-mitotic neurons have similar highly-stable NHOS [21]. The present work expands such result by showing that continued stabilization of the NHOS occurs in post-mitotic neurons even after the fourth post-natal week when according to microanatomical criteria they formally become TD [22]–[24]. The fact that the continued stabilization of the neuronal NHOS as a function of time has no overt impact on gene expression in neurons (Fig. 5), suggests that this phenomenon do not depends on functional constraints.

The DNA of each chromosome constitutes a continuous double-stranded fibre in which each strand has a rigid helical backbone resulting from the strong phosphodiester bonds between the deoxyribose sugars of thousands of nucleotides along the strand, whereas the weak hydrogen bonds between the nitrogenous bases in the anti-parallel strands can be broken and re-established quite easily. Thus the torsional stress of the long DNA molecule along its axis might be dissipated by breaking the hydrogen bonds between both strands, yet by looping and supercoiling along its axis DNA can dissipate the stress without compromising its structural integrity [35]. Hence the interactions DNA-NM that result in a large number of structural DNA loops in the interphase nucleus may be a natural answer to a structural stress problem posed by the intrinsic configuration of DNA [56]. This phenomenon is independent of proteins that constitute chromatin and apparently depends on DNA-NM interactions by means of so-called indirect readouts [46], [47]. Nevertheless, the local chromatin conformation may have a role in determining the choice of DNA sequences available for interaction with the NM in vivo, since chromatin proteins may compete or hinder such DNA-NM interaction.

A DNA loop pattern in which most genes lie close to the actual MARs attached to the NM is observed in baby and young neurons (Fig. 4 and Table 5), similarly to what was reported in hepatocytes of equivalent age [20], but this is a highly improbable distribution considering that protein-coding genes are rare within the genome and so most potential MARs are not close to any protein-coding gene [57]. Yet in the hepatocytes this anomalous distribution changes in time to one in which most genes are distal to the NM, this correlates with a trend towards shortening and homogenization of the average DNA- loop size as a function of age [20]. In aged neurons most target genes studied also become distal to the NM (Fig. 4 and Table 5) indicating that such a trend towards a loop distribution in which genes become distal to the NM as a function of time also holds for neurons, despite the fact that neurons have a rather short and homogeneous average DNA-loop size since early post-natal ages [21]. The gradual stabilization of the NHOS in hepatocytes correlates with their gradual loss of proliferating potential [20]. Nevertheless, the trend towards stabilization of the NHOS with age is observed in both hepatocytes [20] and post-mitotic neurons despite the fact that neurons already have a very stable NHOS from early post-natal age [21]. This fact strongly suggests that thermodynamic and structural constraints drive the NHOS towards maximum stability in time, as the DNA proceeds to dissipate any residual structural stress independently of any cellular functional need [50]. Moreover, considering that entropy is not a measure of disorder or chaos, but of energy diffusion, dissipation or dispersion in a final state compared to an initial state [58], a rather even distribution of short DNA loops anchored to the NM satisfies the second law of thermodynamics, since the structural stress along the DNA molecule is more evenly dispersed within the nuclear volume by increasing the number of stable DNA-NM interactions [50]. The increase in the relative proportion of total DNA actually embedded within the NM (Fig. 3) is evidence that DNA-NM interactions augment in time leading to further stabilization of the NHOS. The fact that several genes located on different chromosome territories within the neuronal nucleus lose in time their original privileged position close to the NM, becoming rather distal to it and so they achieve a distribution relative to the NM typical for most random-sequence DNA tracts, indicates that stabilization of the NHOS occurs as a relentless, physical process above any biological constraint.

**Figure 7:**
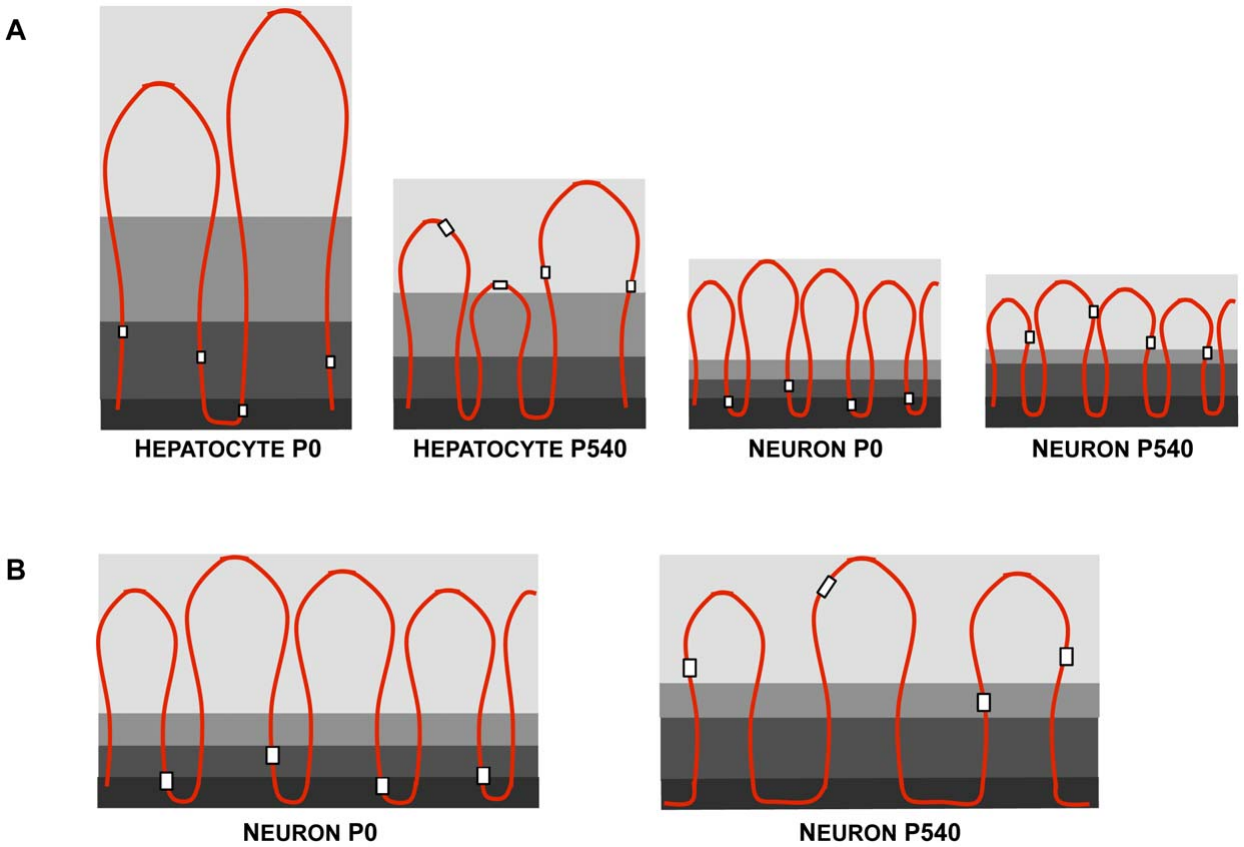
Two possible models for explaining the observed changes in the NHOS as a function of age. A) in newborn rat hepatocytes (P0) with high proliferating potential, the DNA loops are large and heterogeneous in size and yet the target-gene sequences (white squares) are located rather close to the NM (dark grey), while in the aged hepatocytes (P540) DNA loops become shorter, more homogeneous in size as further potential MARs become actualized substituting some previous MARs bound to the NM and so genes become rather distal to the NM, correlating with the significant loss of proliferating potential in aged hepatocytes (hepatocyte-related figures based on our previous data from reference 20). In newborn (P0) post-mitotic neurons the DNA loops are already quite homogeneous and significantly shorter than in aged hepatocytes. Yet in the aged (P540) neurons most of the former MARs bound to the NM have been substituted by other MARs located on average far from any gene, resulting in a significant change in the relative position to the NM of most genes without affecting the average DNA loop size. B) In this case larger stretches of DNA adjacent to the original MARs in P0 neurons, become attached to the NM as a function of time, leading to a reduction in the actual number of DNA loops without significantly affecting their average size but causing a displacement of most genes towards regions distal to the NM. The evidence that a larger fraction of DNA directly interacts with the NM in P540 neurons when compared with P0 neurons (Fig. 3) and that quantitative changes in the NM composition occur with age (Fig. 6) is consistent with the model in B for the case of neurons.

The Fig. 7 depicts two models that may explain how the genes formerly close to the NM become distal to it as a function of time. In the first model (A) genes become distal to the NM because new MARs located far from the genes become actualized substituting in the NM the previous MARs that were close to the genes. Although something like this may be occurring in the case of hepatocytes [20] it is unlikely for the case of neurons considering that there is no real significant change in the already short average DNA-loop size in neurons as a function of age (Fig. 2). However the biochemical data indicate that in P540 neurons a significantly larger fraction of total DNA is embedded in the NM when compared with the corresponding fraction in neurons from earlier ages (Fig. 3). This fact is consistent with the second model in Fig. 7 (B) which suggests that the actual length of each established MAR increases as a function of time because more DNA adjacent to the original MAR becomes directly bound to the NM. This results in an overall lesser number of DNA loops with the corresponding displacement of genes to regions relatively distal from the anchoring points to the NM, without significantly changing the average DNA loop size. This model is consistent with our data and with evidence for the existence of rather lengthy MARs [59], but we cannot directly measure any comparative change in the average MAR length as a function of time since for that purpose we need to destroy the NM in order to liberate the bound MARs, a procedure that leads to unavoidable fragmentation of the formerly bound MARs. Yet, the quantitative changes in the composition of the neuronal NM as a function of age (Fig. 6) parallel those previously described in aged hepatocytes [20] and may explain how the conditions that allow to increase the direct interactions between DNA and the NM appear in time.

Early death is observed in neurons forced to re-enter the cell cycle [8] and neuronal cell cycle activity has been observed early in several diseases that course with neurodegeneration [7]. Reactivated post-mitotic TD cells such as myotubes die very quickly, from apotosis, after re-entry into the cell cycle. The apoptotic process is triggered by significant DNA damage resulting from the attempted DNA replication and those few myotubes that proceed to mitosis show aberrant mitotic spindles and other serious mitotic anomalies before dying [2]. DNA is replicated in macromolecular complexes know as replication factories organized upon the NM [60], [61]. Varied evidence indicates that DNA loops correspond to the actual replicons in vivo [44], [61]–[64]. Thus, considering that highly stable physical systems are resistant to change and have a much reduced dynamic potential, resulting from intrinsically high activation-energy barriers, it is relatively easy to imagine scenarios in which forced replication in post-mitotic cells having a highly stable NHOS leads to severe replicon (DNA) damage and cellular death, as it has been observed [2].

A structurally-stable cell nucleus is unlikely to be the seat of both the dynamic transitions necessary for mitosis and the rearrangement of chromosome territories and chromatin domains that occurs in early G1 [65], since it is conceivable that under such conditions the high activation-energy cost of mitosis, that includes chromosome condensation and nuclear disassembly, would be limiting for the cell. Indeed, polyploidy is a common feature of senescent cells and post-mitotic cells [66], suggesting that some intrinsic factor prevents such cells, endowed with a functional DNA-replication machinery, from achieving mitosis.

Nuclear reprogramming by nuclear transfer (NT) is a tool for testing functionally nuclear potency and for distinguish genetic from epigenetic alterations in the donor nucleus. Cloning of animals by NT is extremely inefficient and the more differentiated the donor cell is the less likely the success of the NT [67], [68]. This fact indicates the presence of strong epigenetic constraints in TD cells, although in the current literature the nature of such constraints is reduced to a matter of DNA methylation and biochemical modifications in histones and other chromatin proteins, without considering the changes in NHOS. Interestingly, cloning of mice using as donors nuclei from neural cells from the adult cerebral cortex was unsuccessful [69] and it has been reported that the developmental potency of neural cell nuclei is dramatically reduced as the cells migrate from the immature neural stage in the ventricular (V) zone to the post-mitotic stage in the pial (P) zone of the developing cerebral cortex. The marked difference between nuclei from the V and P zone with respect to their ability for supporting normal embryonic development, led to the suggestion that some dramatic changes occur within the nuclei of the differentiating neural cells [70]. However, the successful cloning of mice using nuclei from post-mitotic olfactory sensory neurons has been achieved and demonstrates that the mechanisms causing the exit of the cell cycle and irreversible mitotic arrest in post-mitotic neurons can be reverted in the environment of the egg [71]. The cloning of mice using nuclei from post-mitotic olfactory sensory neurons also provided evidence that neuronal differentiation is not dependent on irreversible rearrangements at the level of DNA sequence [71], yet in the present work we show that significant rearrangements of the neuronal NHOS occur throughout post-natal development without affecting the DNA sequence (Fig. 4 and Table 5).

Successful nuclear reprogramming following somatic cell NT into a egg cytoplast depends on dramatic morphological and biochemical changes in the NHOS of the donor nucleus that result in nuclear remodeling characterized by breakdown of the nuclear envelope, disassembly of the nuclear lamina, dispersion of nucleoli, premature chromosome condensation, and loss of nuclear lamins A/C whose expression is characteristic of differentiated cells but not of pluripotent or early-differentiating cells [72]–[74]. About how the nuclear remodelling is achieved we can only speculate. Yet it is worth considering that the oocyte is the cell with the largest number of mitochondria, at least an order of magnitude greater than the number in somatic cells [75]. Hence in the oocyte there is a surplus of energy that may be able to overcome high-activation energy barriers.

In some cell types like hepatocytes the stabilization of the NHOS occurs slowly throughout post-natal life and this correlates with loss of proliferating potential [20]. However, P0 and P7 cortical neurons are already post-mitotic and their NHOS is even more stable than that in aged hepatocytes [21 and this work]. This fact suggests that cellular proliferating potential is permanently lost once the NHOS reaches stability beyond a certain threshold [50]. Heterochrony during development may explain why some cell types achieve that critical stability of their NHOS that precludes any further cell division, before other cell types. Nevertheless, in vivo the NHOS may keep evolving in time towards further stability provided there is any remaining structural stress in the DNA worth of being dissipated [21], [50].

Overall our results are consistent with a model in which the NHOS of neurons constitutes an integral structural system that naturally but relentlessly evolves in time towards a highly-stable state obeying thermodynamic constraints [50] establishing, after crossing a certain stability threshold, an insurmountable high-activation energy barrier to mitosis that guarantees the structural, non-reversible nature of the neuronal post-mitotic state.

## Materials and Methods

### Animals

Male Wistar rats were used for isolation of rat hepatocytes and neuronal nuclei. All animals were used in accordance with the official Mexican norm for production, care and use of laboratory animals (**NOM**-062-ZOO-1999). Protocol (48447-Q) approved by the Bioethics Committee of the School of Medicine of the Universidad Autónoma del Estado de México.

### Hepatocytes

Primary rat hepatocytes were obtained from male rat livers, using the protocol previously described [37]. Briefly, the livers were washed in situ by perfusion with PBS without Ca^2+^ and Mg^2+^ (PBS-A) at 37°C for 5 min at 15 ml/min. Next the tissue was perfused with a solution of collagenase IV, Sigma (0.025% collagenase with 0.075% of CaCl_2_ in Hepes buffer, pH 7.6) for 8 min. Viable hepatocytes were counted in a haemocytometer and used immediately for preparing the nucleoids.

### Preparation of nucleoids from hepatocytes

Nucleoids were prepared as previously described [37], [76]. Briefly, freshly isolated washed hepatocytes were suspended in ice-cold PBS-A, aliquots of 50 µl containing 3.5×10^5^ cells were gently mixed with 150 µl of a lysis solution containing 2.6 M NaCl, 1.3 mM EDTA, 2.6 mM Tris, 0.6% Triton-X 100 (pH 8.0). After 20 min at 4°C, the mixture was washed in 14 ml of PBS-A at 4°C for 4 min at 3000 rpm (1500 g). The pellet was recovered in a volume ranging from 200 to 300 µl.

### Neuronal nuclei isolation

Neuronal nuclei from cerebral cortex of rats were isolated as described by Thompson [25] with some modifications. The cerebral cortex was dissected from the rat brain and it was homogenized with 1 ml of 2.0 M sucrose (1 mM MgCl_2_+0.25 mM phenylmethylsulfonyl fluoride). The homogenate was transferred to a fresh tube containing 5 ml of 2.0 M sucrose (1 mM MgCl_2_+0.25 mM PMSF) forming a two phase suspension. The suspension was spun at 4°C for 60 min at 49,000 g in a KR 25i centrifuge. In the case of P0 samples the pellet was washed with 10 ml of 0.32 M sucrose (1 mM MgCl_2_+0.25 mM PMSF) and spun for 5 min at 1500 g. The P0 pellet was resuspended in 1 ml of 0.32 M sucrose. For P7, P80 and P540 samples the pellet was resuspended in 4 ml of 2.4 M sucrose (1 mM MgCl_2_+0.25 mM PMSF) and a gradient was formed by adding 2 ml of 1.8 M sucrose (1 mM MgCl_2_+0.25 mM PMSF) that was centrifuged at 4°C for 60 min at 49,000 g. From this centrifugation it is obtained a fraction of nuclei in the interphase between 2.4 M and 1.8 M sucrose (N1) and a further fraction of nuclei in the pellet (N2). Each nuclei population (N1 or N2) was separately washed in 10 ml of 0.32 M sucrose (1 mM MgCl_2_+0.25 mM PMSF) and spun for 5 min at 1500 g. The isolated neuronal nuclei were counted in a haemocytometer and used immediately for preparing nucleoids.

### Preparation of nucleoids from neuronal nuclei

Freshly isolated neuronal nuclei were suspended in 0.32 M sucrose and aliquots of 50 µl containing 5×10^5^ nuclei were gently mixed with 150 µl of lysis solution (2.6 M NaCl, 1.3 mM EDTA, 2.6 mM Tris, 0.6% Triton-X 100, pH 8.0). After 10 min at 4°C a small aliquot was stained with ethidium bromide (EB) 80 µg/ml and examined by fluorescence microscopy for assessing their integrity and DNA-loop supercoiling [75]. The mixture was washed with 14 ml of (PBS-A) at 4°C for 5 min at 1500 g. The nucleoids pellet was recovered in a volume of 200 to 300 µl.

### Identification of nuclei and nucleoids from neurons

In nuclei and nucleoid preparations obtained from cells of rat cerebral cortex the neuron specific nuclei and nucleoids were detected using a monoclonal mouse antibody against the neuron-specific nuclear protein NeuN (Chemicon). 100 µl of freshly isolated nuclei or nucleoids were mounted and dried onto gelatine-coated slides for 90 min at room temperature. Mounted nuclei or nucleoids were fixed with ice-cold acetone for 10 min and washed three times in ice-cold PBS-A. Non specific binding sites were blocked with 10% non-fat skimmed milk powder in PBS-A overnight, prior to incubation with anti-NeuN antibody (1∶500) in PBS-A containing 0.01% Tween 20 at 4°C for 2 h. After washing three times in PBS-A for 5 minutes, the samples were incubated with anti-mouse-Rhodamine antibody (1∶1000, ICN Pharmaceuticals) in PBS-A at 4°C for 30 min. Next, after washing three times in PBS-A for 5 min the samples were mounted with DABCO and examined by epifluorescence microscopy. Negative controls were performed by omission of the primary antibody. The percentage of neuron specific nuclei and nucleoids was estimated by counting positive-stained nuclei/nucleoids against non-stained (Table 1). The resulting nucleoid preparations are highly enriched (>92%) in neuron-specific nucleoids and so it is possible to use the freshly isolated nuclei from the cerebral cortex for obtaining nucleoid preparations that are highly enriched in neuronal nucleoids.

### DNase I digestion of nucleoids

The washed neuronal nucleoids were pooled (2.3×10^6^ in 1.2 ml PBS-A) for setting up the DNase I digestion curves and mixed with 5.1 ml of DNase I digestion buffer containing 10 mM MgCl_2_, 0.2 mM 2-Mercaptoethanol, 50 mM Tris at pH 7.2. The digestions were carried out at 37°C with 0.92 U/ml DNase I (Sigma) from a stock (0.15 M NaCl, 50% Glycerol, 2,300 U/ml DNasa I). For each digestion time point an aliquot of 1 ml containing 3.6×10^5^ nucleoids was used and the digestion was stopped by adding 200 µl of stop buffer containing 0.2 M EDTA, 10 mM Tris at pH 7.5. After digestion the samples were spun for 10 min at 9000 g at 4°C. The pellet was washed once with 1 ml of PBS-A, stirred, and centrifuged for 10 min at 9000 g at 4°C, washed twice with 1 ml of ice-cold double-deionised water (dd-H_2_O) and centrifuged again as above. The final pellet was resuspended in 100 µl of dd-H_2_O to be used directly as template for PCR. The DNA amount present in samples from each digestion time-point was determined by spectrometry.

### PCR amplification

Nine short amplicons (target sequences) corresponding to eight different genes located on six separate chromosomes: *NFM* (15p12); *NFL* (14); *MBP* (18 q11–q13); *GFAP* (10q32.1); *MPZ5′* (13q24–q25); *MPZ3′* (13q24–q25); *ACT* (12q11); *ALB* (14p22); *AFP* (14p21), were designed for mapping their position relative to the NM in neuronal nucleoid preparations (Table 4).

Standard PCR was carried out using 1.25 U GoTaq Flexi DNA polymerase (Promega) and 60 ng of nuclear matrix-bound DNA obtained from each DNase I digestion point, using an Applied Biosystems 2720 thermal cycler, and the same amplification program was used for all pairs of primers (Table 1): initial denaturising step at 94°C for 5 min, denaturising step at 94°C for 45 s, annealing at 56°C for 30 s, and extension at 72°C for 1 min for 35 cycles, with a final extension at 72°C for 10 min. The identity of all the amplicons was confirmed by restriction analysis with the appropriate restriction enzymes. Amplicons were electrophoresed on 2% agarose gels and visualized using EB staining (0.5 µg/ml), recorded and analyzed using a Kodak 1D Image Analysis Software 3.5 system. Amplicons were scored as positive or negative on partially digested nucleoid samples, depending on whether they are detected or not by the software using its default settings.

### Reverse transcriptase-PCR analysis of mRNA

50–100 ng of freshly isolated cerebral cortex from either P0, P7, P80 or P540 rats were mixed with 1 ml TRIZOL (GIBCO-BRL) for isolating total RNA according to the manufacturer's instructions. 100 ng of sample-specific RNA were mixed with 1 µl of random primers in a 12 µl volume and incubated at 94°C for 5 min. Next 4 µl of RT buffer (250 mM Tris-HCl pH 8.3, 375 mM KCl, 15 mM MgCl_2_), 2 µl of dithiotreitol 0.1 M, 0.9 µl of reverse transcriptase (40 U/µl SuperScript TM II, GIBCO-BRL) and 0.7 µl of dNTPs 10 mM were added in a final volume of 20 µl. cDNA amplification was achieved with the following program: alignment at 25°C for 10 min, extension at 42°C for 60 min and final extension at 70°C for 15 min. The reaction product was incubated 20 min with 0.8 µl RNase H (2 U/µl, Invitrogen) at 37°C. The resulting cDNA was used as template for PCR amplification of the appropriate gene-specific amplicons (Table 4), following the previously described program for PCR amplification.

### SDS-PAGE

Neuronal nucleoids from rats of different post-natal ages were digested with DNase I 30 U/ml for 3 h at 37°C. Digested-nucleoid aliquots were centrifuged at 9,000 g 10 min at 4°C. The pellet was washed with PBS-A and dd H_2_O. Next all washed pellets were pooled and further washed in dd H_2_O. The final pellet was homogenized in 100 µl of buffer for extraction of proteins and boiled for 5 min. Extracted proteins were quantified by spectrophotometry using the DC-protein assay kit (Bio-Rad) and stored at −70°C. NM protein profiles were obtained following standard SDS-PAGE in 10% polyacrylamide gels. Each lane was loaded with 15 µg of protein. Gels were run at 40 mA and stained with Coomassie blue for analysis with a Kodak 1D Image Analysis Software 3.5

### Fluorescence microscopy

All procedures involving epifluorescence microscopy were carried out using an Olympus BX60 microscope fitted with an Evolution MP Colour camera for image and video acquisition. Images and videos were processed with the Image-Pro Plus digital image analysis system Version 4.5 (Media-Cybernetics).

### Nucleoid fluorescent DNA halo assay

Hepatocyte or neuronal nucleoids aliquots (10 µl) were deposited directly on a slide and stained with 10 µl of ethidum bromide at 160 µg/ml (final concentration 80 µg/ml) and examined by fluorescence microscopy after 30 s of DNA-halo expansion for assessing the nucleoid DNA integrity and supercoiling [76], [77]. Average DNA halo radii were estimated from the core nucleoid contour to the outer limit of the DNA halo using the Image-Pro-Plus image-analysis software. For real-time nucleoid videos, selected representative nucleoids were focused under phase contrast and a sequence of 30 fluorescent images was captured using the minimum possible time interval.

### Nuclei and nuclear matrix measurements

The diameter (D, expressed in µm) of either isolated nuclei or nuclear matrix phase-contrast images was measured using the Image-Pro-Plus image-analysis software.

## Supporting Information

Video S1 Nucleoid fluorescent DNA halo assay: real-time video clip showing a P0 rat neuron nucleoid exposed to 80 µg/ml of ethidium bromide. Notice the unwinding of DNA loops that form a very homogeneous but rather small DNA halo around the contour of a very stable NM.
(AVI)

Video S2 Nucleoid fluorescent DNA halo assay: real-time video clip showing a P7 rat neuron nucleoid exposed to 80 µg/ml of ethidium bromide. Notice the unwinding of DNA loops that form a very homogeneous but rather small DNA halo around the contour of a very stable NM.
(AVI)

Video S3 Nucleoid fluorescent DNA halo assay: real-time video clip showing a P80 rat neuron nucleoid exposed to 80 µg/ml of ethidium bromide. Notice the unwinding of DNA loops that form a very homogeneous but rather small DNA halo around the contour of a very stable NM.
(AVI)

Video S4 Nucleoid fluorescent DNA halo assay: real-time video clip showing a P540 rat neuron nucleoid exposed to 80 µg/ml of ethidium bromide. Notice the unwinding of DNA loops that form a very homogeneous but rather small DNA halo around the contour of a very stable NM.
(AVI)

Video S5 Nucleoid fluorescent DNA halo assay: real-time video clip showing a P0 rat hepatocyte nucleoid exposed to 80 µg/ml of ethidium bromide. Notice the complete unwinding and release of DNA loops and the simultaneous complete disintegration of the NM.
(AVI)

Video S6 Nucleoid fluorescent DNA halo assay: real-time video clip showing a P7 rat hepatocyte nucleoid exposed to 80 µg/ml of ethidium bromide. Notice the complete unwinding and release of DNA loops and the simultaneous fragmentation of the NM.
(AVI)

Video S7 Nucleoid fluorescent DNA halo assay: real-time video clip showing a P80 rat hepatocyte nucleoid exposed to 80 µg/ml of ethidium bromide. Notice the unwinding of DNA loops that form a very homogeneous DNA halo around the contour of a stable NM.
(AVI)

Video S8 Nucleoid fluorescent DNA halo assay: real-time video clip showing a P540 rat hepatocyte nucleoid exposed to 80 µg/ml of ethidium bromide. Notice the unwinding of DNA loops that form a very homogeneous but rather small DNA halo around the contour of a larger and very stable NM.
(AVI)
